# F2. CHILDHOOD TRAUMA AND LACK OF CULTURAL IDENTITY AS RISK FACTORS OF ATTENUATED PSYCHOSIS SYMPTOMS AMONG AFRICAN AMERICAN YOUNG ADULTS

**DOI:** 10.1093/schbul/sby017.533

**Published:** 2018-04-01

**Authors:** Huijun Li, Monique Rowe

**Affiliations:** 1 Florida A&M University; 2 Jewish Community Center

## Abstract

**Background:**

Schizophrenia spectrum diagnosis is more commonly assigned to African Americans. Failing to understand and appropriately manage cultural differences will have significant mental health consequences for varied racial/ethnic groups in particular (Betancourt, Green, & Carrillo, 2002). The purpose of the present study was to examine risk factors of attenuated psychosis syndrome in a sample of African American young adults, specifically to investigate whether lack of ethnic identity and adverse childhood experiences (ACEs) put an individual at a higher risk of developing attenuated psychotic symptoms.

Adverse Childhood Experiences (ACE) as Risk Factor of APS:

The Comorbidity Survey (NCS) Part 2 data showed that the effects of neglect and sexual abuse, along with physical abuse similarly put a child at risk for psychosis. People who had suffered childhood adversity were 2.8 times more likely to develop psychosis than those who had not. Studies have also begun to look at gender differences in schizophrenia by way of ACEs.

Lack of Ethnic Identity as Risk Factors of APS:

The African worldview reflects psychological communal, spiritual, collective survival thrust as opposed to the European worldview of individualism and materialism. Cultural Misorientation (CM) represents that foreign psychological or psychopathological disposition in the African personality, which allows African Americans to unknowingly value and participate in European cultural indoctrination through the practice of European cultural values, rituals, and customs. The purpose of this study was to explore the roles that CM play on the overall presentation of attenuated psychotic symptoms, by way of ACE exposure.

**Methods:**

Participants: Participants included 304 African American college students, 199 (65.46%) women and 105 (34.54%) men from a Historically Black College and University in the southeastern region of the United States. Participants were between 18 and 25 years of age.

**Instruments:**

Adverse Childhood Experiences Scale measures the association of multiple types of abuse with different types of health outcomes. Prodromal Questionnaire- Brief (PQ-B) measures the presence of negative symptoms, perceptual abnormalities such as hallucinations, and unusual thought content like delusional ideas and paranoia. Cultural Misorientation - Short Form assesses the condition of cultural misorientation across 6 subscales-- materialism orientation, individualism orientation, alien-self orientation, anti-self orientation, self-destructive orientation, and integration orientation.

**Results:**

The Pearson correlation analysis indicated no significant relationship (r = -.073, p = .206) between ACE exposure and APS total scores on PQ-B. However, an unexpected negative significant relationship between childhood abuse exposures and symptom severity was observed (r = -.126*, p = .028), indicating that participants who reported more instances of childhood abuse tended to report less symptom severity. In addition, Cultural Misorientation (CM) was significantly positively correlated to PBQ total scores r = .194**, p = .001) and the severity of those symptoms (r = .171**, p = .003). CM materialism and individualism subscales mediated the relationship between childhood abuse and PQ-B total scores and symptom severity.

**Discussion:**

This study provides support that some aspects of cultural misorientation can be detrimental to African Americans. Helping to reduce material and individualistic desires that have become detrimental should also be a central focus of implemented mental health programs.

